# Cortical neurons of bats respond best to echoes from nearest targets when listening to natural biosonar multi-echo streams

**DOI:** 10.1038/srep35991

**Published:** 2016-10-27

**Authors:** M. Jerome Beetz, Julio C. Hechavarría, Manfred Kössl

**Affiliations:** 1 Institut für Zellbiologie und Neurowissenschaft, Goethe-Universität, Frankfurt/M., Germany

## Abstract

Bats orientate in darkness by listening to echoes from their biosonar calls, a behaviour known as echolocation. Recent studies showed that cortical neurons respond in a highly selective manner when stimulated with natural echolocation sequences that contain echoes from single targets. However, it remains unknown how cortical neurons process echolocation sequences containing echo information from multiple objects. In the present study, we used echolocation sequences containing echoes from three, two or one object separated in the space depth as stimuli to study neuronal activity in the bat auditory cortex. Neuronal activity was recorded with multi-electrode arrays placed in the dorsal auditory cortex, where neurons tuned to target-distance are found. Our results show that target-distance encoding neurons are mostly selective to echoes coming from the closest object, and that the representation of echo information from distant objects is selectively suppressed. This suppression extends over a large part of the dorsal auditory cortex and may override possible parallel processing of multiple objects. The presented data suggest that global cortical suppression might establish a cortical “default mode” that allows selectively focusing on close obstacle even without active attention from the animals.

Animals are continuously exposed to multiple stimuli and their sensory systems should selectively respond to behaviourally-relevant information. Selective attention^1^, alternation of fixation points through saccadic eye movements in the visual system^2^, detection of rarely occurring stimuli^3^,^4^,^5^ and habituation, are some strategies that can account for selective neuronal responses to behaviourally relevant information.

Echolocating bats predominantly rely on their auditory system for short range orientation. Bats broadcast sequences of echolocation calls and they extract relevant orientation cues from echoes that are reflected from surrounding objects^6^,^7^,^8^,^9^. Behavioral studies have shown that bats can alternate the direction of their calls. Thus, their focus changes between objects in a saccadic manner resembling saccadic eye movement^6^,^10^. However, at present, it remains uncertain to which extent bats can spatially restrict their sonar beam. Especially when multiple objects are arranged sequentially at different space depths, a single call could still result in multiple echoes that arrive one after the other to the bats’ ears. The mechanisms by which bats can extract object distance information from such natural and complex multiple-object sequences remain obscure.

Bats calculate the object distance with the aid of the echo-delay, which is the time elapsed from emitting a call until the arrival of the corresponding echo. The smaller the echo-delay the shorter the distance to an object. Within the ascending auditory pathway, neurons whose response is facilitated when presented with specific echo-delays can be found as early as in the auditory midbrain^11^ and these neurons are defined as target-distance or delay-tuned neurons.

In this study, it is investigated how delay-tuned neurons in the auditory cortex of the fruit-eating bat *Carollia perspicillata* respond to distance information from multiple objects. The main goal was to test whether neuronal responses to multiple-object sequences can be predicted from responses to single-object sequences, created by deleting object-specific echoes in the multiple-object sequences. To achieve this goal we recorded from anesthetized animals while using an echolocation sequence that contained information from multiple echoes as stimulus. The results indicate that only certain portions of the response to multiple-object sequences can be predicted accurately from responses to single-object sequences. More specifically, only responses to leading echoes, which originate from the nearest object, are comparable to responses elicited by the same echo in single-object sequences. Responses to temporally lagging echoes that indicate distant targets are suppressed. It is suggested that such selective echo-suppression could be important for “default focussing” of neuronal responses on nearest obstacles. The animals were anaesthetized during the experiments which emphasizes that the selective suppression is a rather basic cortical feature and acts independent of active attentional mechanisms. Processing distance information from nearest objects could be ethologically crucial for evoking fast motor responses for obstacle avoidance during flight.

## Results

### Neuronal response of echo-delay tuned neurons to multiple-object sequences

**Figure 1:**
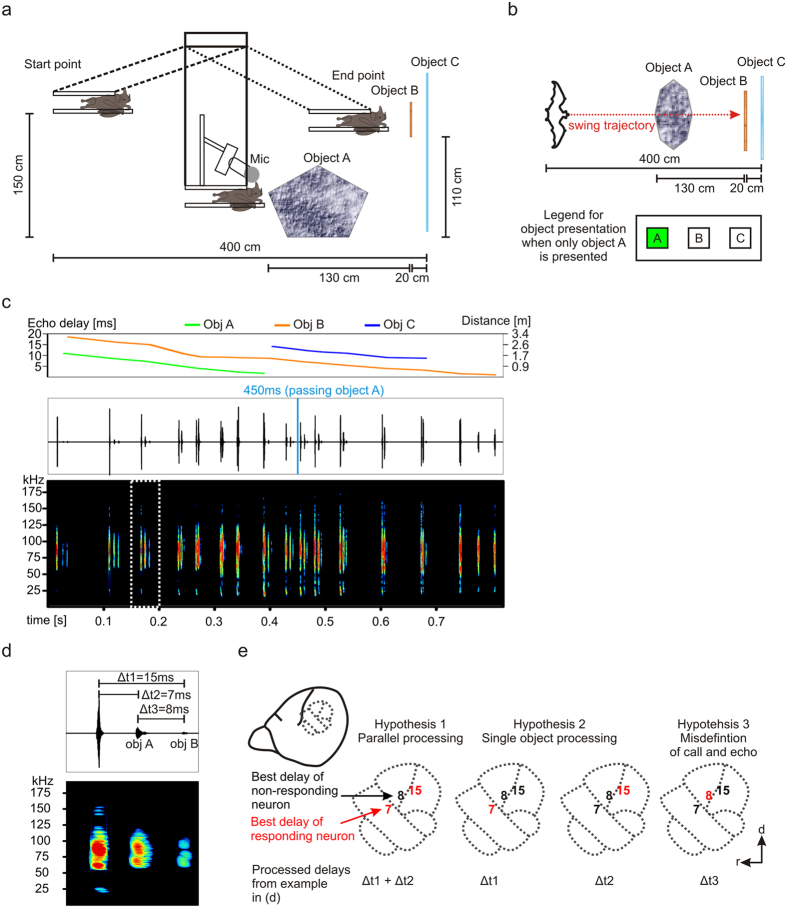
Natural echolocation sequence consisting echo information from three objects. (**a**) Schematic side view (not in scale) of the pendulum paradigm. The animal was fixed in a pendulum and it was swung towards three objects. During the swing the animal echolocates. Biosonar calls and echoes were recorded with an ultrasonic microphone (Mic) that was positioned above the animal’s head and it was pointing into the direction of the swing. The distance between the microphone and the animal’s ears was about 4 cm. During the swing object A, mimicking the shape of a rock was overflown by the bat. Object B was a wooden plate and object C was an acrylic glass wall. (**b**) Top: Schematic top view (not in scale) of the recording set up. Bottom: Example of a legend indicating a “single-object sequence” in which object A (green filled rectangle) is represented in the echolocation sequence, while echoes from object B and object C (unfilled rectangles) are absent. (**c**) Top: Change of object specific “echo-delays” along the time axis of the sequence. Each object is represented by a differently colored line. Bottom: Oscillogram and spectrogram of the echolocation sequence that was used as acoustic stimulus for electrophysiological recordings. The blue vertical line at 450 ms indicates the time point of passing object A. (**d**) Magnification of one call-echo element marked by white dashed rectangle in (**c**). The echo-delays are indicated in the oscillogram. (**e**) Three hypotheses (1–3) regarding how the echo-delays of the call-echo element in (**d**) could be processed in the cortex of *C. perspicillata*. Schematic lateral view on *C. perspicillata*’s brain and magnified auditory cortical areas (dashed lines). Numbers in the cortical areas represent the best delay to which the neurons respond to. Red numbers indicate responding and black numbers non-responding neurons when the animal is stimulated with the call-echo element based on the corresponding hypothesis. Note that according to hypothesis 3 the delay between two consecutive echoes (Δt3 in **d**) is processed and not the echo-delay between call and echo (Δt1 or Δt2 in **d**). *d* = dorsal; *r* = rostral.

For recording a natural multiple-object sequence, a bat was positioned in a pendulum (method after refs 12, 13, 14) and it was swung towards three different objects that were separated along the depth axis (Fig. 1a,b). To limit the complexity of the echo spectra three well reflecting objects with smooth surfaces were chosen. Object A had a rock-like shape and it was built out of papier-mâché. Object B was a wooden plate and object C was an acrylic glass wall. Object A was overflown by the animal at about 450 ms after the start of the swing (Fig. 1c, blue vertical line) before the swing stopped directly in front of object B which was positioned 130 cm behind object A. Object C was placed 20 cm behind object B. Object C was larger in height and width than object B, to ensure that object B was not entirely overlapping object C in space and that a faint echo from object C could be detected after the echo of object B.

The broadcasted calls and their echoes, reflected from the three objects, were recorded with an ultrasonic microphone located on top of the animal at 4 cm distance to both ears. The multiple-object sequence consisted of 17 biosonar calls in which each call elicited at least two echoes coming from two different objects (Fig. 1c). Echo-delays that were elicited by call reflections from object A ranged between 11 and 1.7 ms (Supplementary Table 1). Echoes from object B were continuously represented throughout the swing with delays ranging from 18.6 to 1 ms. Echo information from object C covered echo-delays from 14.2 to 8.7 ms. Echoes from object C were elicited as early as the 400 ms after the start of the swing. Note that the late occurrence of echoes from C can be related to the fact that during the first portion of the swing, this object was simply too far away for eliciting echoes that were intense enough for being recorded. Also, object A spatially overlapped with object C, thus, calls that were spatially focused on object A likely could not reach object C. At about 700 ms after the start of the swing, object B spatially overlapped with object C, thus, in this situation no biosonar call was reflected from object C. To explore the impact of each object on cortical processing, single-object-sequences and dual-object sequences were created by deleting object-specific echoes from the multiple-object sequence. The sequences were used as acoustic stimuli in electrophysiological recordings from anaesthetized animals. Note that anaesthesia should prevent attention-dependent effects on the neuronal response.

Regarding the multiple-object sequences, when the bats are stimulated with a call and two echoes coming from two different objects (Fig. 1d), delay-tuned neurons could respond in different ways as illustrated in Fig. 1e.Two different neuron types according to their best delay, could process distance information from both objects (Hypothesis 1 in Fig. 1e). One neuron tuned to 7 ms delay (indicated in red in Fig. 1e) responds to the leading echo and a second neuron tuned to 15 ms delay responds to the lagging echo. Another processing strategy would be that the neuronal population predominantly respond to echoes coming from a single object that produces either leading or lagging echoes (Hypothesis 2). In such a scenario, neurons tuned to a specific echo-delay would respond accordingly to a certain object but the activity of other neurons that could theoretically respond to echo-delays coming from other objects is reduced. In the behaviourally worst case, neurons would respond to the delay between leading and lagging echoes (processing the distances between objects; Hypothesis 3). This processing strategy could lead to misinterpretations since the first echo would be mistaken as a call.

**Figure 2:**
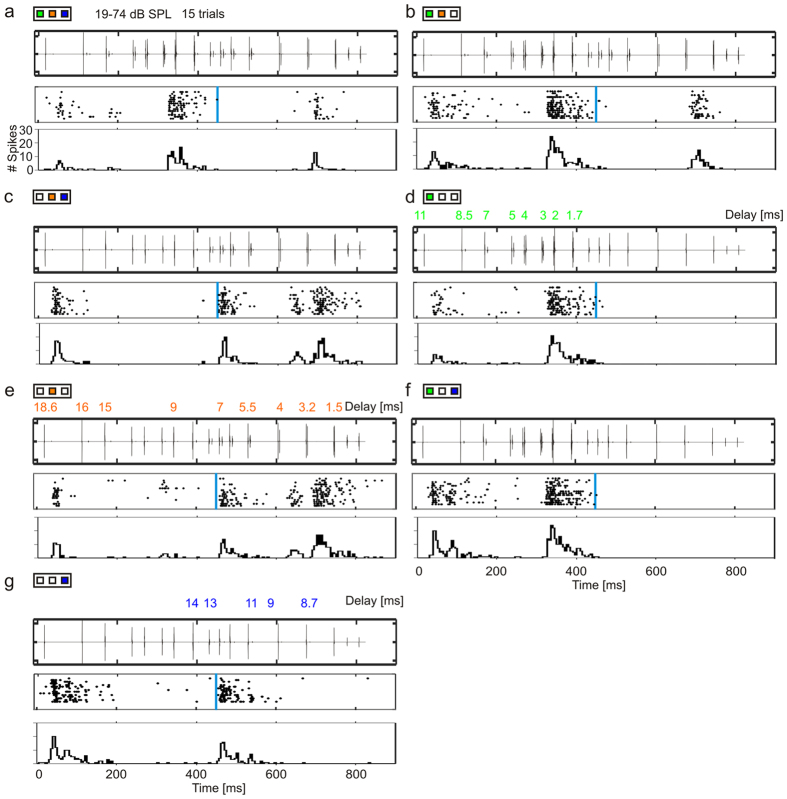
Responses to multiple- and to different single- and dual-object sequences. For each stimulus condition the oscillogram of the acoustic sequence (top plot), the raster plot, and post-stimulus time histogram (PSTH; binsize = 5 ms) of the neuronal response from a multi-unit is shown (middle and bottom plots). Vertical blue lines indicate the time point where echoes from object A are absent due to the bat leaving that object behind in the flight trajectory. Colored numbers above the oscillograms in (**d**,**e**,**g**) indicate some delays that are presented at that particular time point of the corresponding sequence. (**a**–**g**) Response to object ABC (**a**), AB (**b**), BC (**c**), A (**d**), B (**e**), AC (**f**), and C (**g**) sequences. Note that after passing object A, stimulations in (**a**,**c**,**b**,**e**) as well as stimulations in (**f**,**g**) are the same.

The three possibilities mentioned in the preceding text were tested by analysing neuronal responses from 96 units. Figure 2a–g shows a neuronal response to the sequences consisting of echo information from combinations of objects A, B, and C. The neuronal activity of the same unit in response to other intensity levels is shown in supplementary Figure 1. Delay tuning was assessed based on the time point of the maximum response (best delay) in response to the echolocation sequence that contained echoes from object B only (B sequence; Fig. 2e). The B sequence was used for determining the best delay because that single-object sequence covered the largest delay range from 1 ms–18.6 ms (orange plot in Fig. 1c, *top*). The unit was tuned to short-delays and during the presentation of sequence A and sequence B responded best at 2–3 ms echo-delays (Fig. 2d,e). In the response to the B and C sequence (Fig. 2e,g) the unit did also respond to echo-delays 7 and 13 ms, respectively. These peaks could arise from the fact that delay-tuning strongly depends on the level relationship between pulse and echo, which strongly varied during the sequences^13^,^15^,^16^,^17^. Usually, the higher the levels are, the wider is the rang of the delay-tuning^14^. Note that in response to the same sequences presented at lower sound pressure levels those peaks in the response were not detected (supplementary Figure 1).

To investigate the impact of echo information from object A on the neuronal tuning, one might can check the neuronal activity in the second half of the sequence beginning from the 450 ms mark. Note that after, the 450 ms mark no echo from object A occurs (indicated by the blue vertical lines in Fig. 2), since at that position the bat had already overflown object A. From that time point on, the multiple-object sequence (Fig. 2a) was equal to the dual-object sequence containing echoes from object B and C (BC sequence; Fig. 2c). The same is true when comparing the AB sequence (Fig. 2b) with the B sequence (Fig. 2e) or the AC sequence (Fig. 2f) with the C sequence (Fig. 2g). Note that both for the ABC versus BC and the AB versus B sequences, the echo composition was the same after the 450 ms mark. Nevertheless, after 450 ms the response to sequences that contained echo information from object A (Fig. 2a,b,f) was weaker than those in the corresponding sequences containing no echo from object A (Fig. 2c,e,g). In other words, it appears that the presence of echoes from object A triggers a suppression that hampers the representation of echoes from more distant objects. Such suppression operates in the time window from 450 ms until the end of the sequence.

### Forward suppression and recovery in response to multiple-object sequences

For the purpose of simplicity, the analysis of suppression described below is based on dual-object sequences. For getting an overview about the suppression in the multiple-object sequence see Supplementary Figure 2. We focussed here on the suppression in the dual-object sequence because responses to such sequences can be directly compared with responses to a single-object sequence where only echo information from one object is processed. Comparing responses to the multiple-object sequence with responses to dual-object sequences could be ambiguous because in dual-object sequences suppression could be caused by the presence of echoes from two objects.

**Figure 3:**
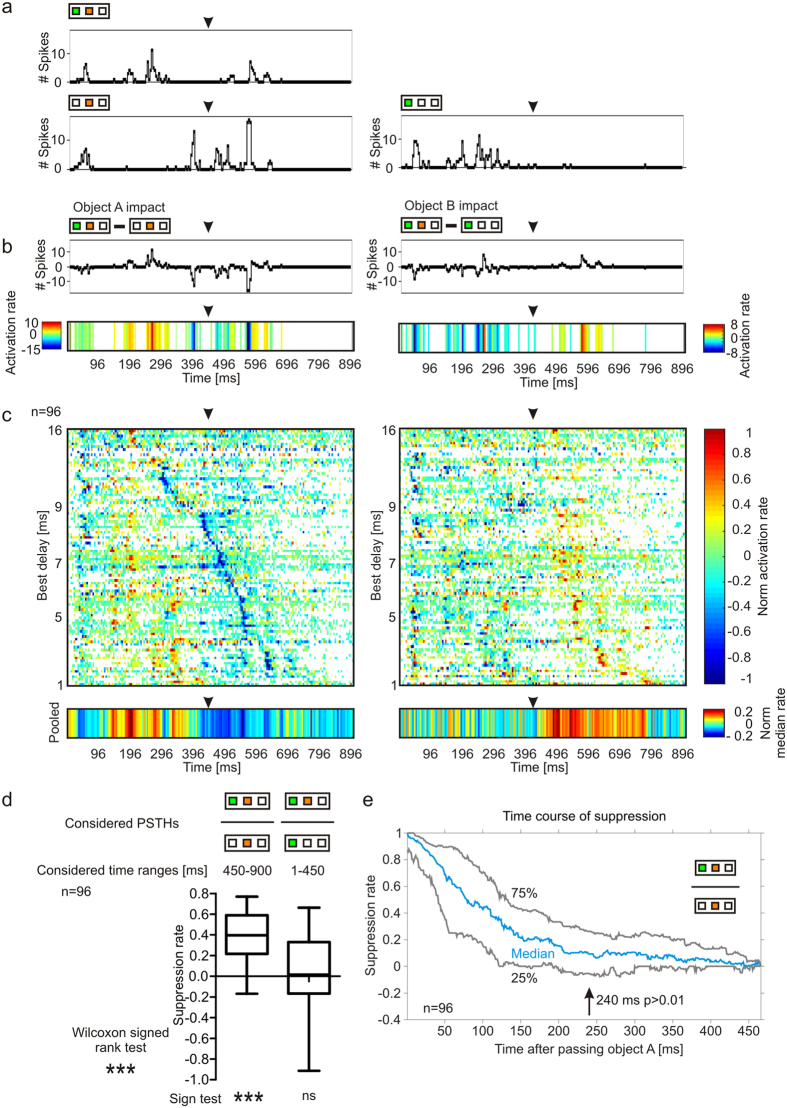
Quantification and time course of suppression when stimulated with a dual-object sequence. (**a**) Top: PSTH in response to the AB sequence. Bottom: PSTHs in response to the B and A sequence. Black arrowheads signal time point of passing object A. (**b**) Left: To investigate the impact of object A on the response to the AB sequence the PSTH to the B sequence was subtracted from the PSTH to the AB sequence. The obtained activation rates are plotted into a line plot and into a color-map. For the color-maps, bins with no difference between dual- and single-object sequences are white. Negative values indicate suppressive and positive values excitatory impacts of the corresponding object on the response to the dual-object sequence. Right: To investigate the impact of object B on the response to the AB sequence, the PSTH to the A sequence was subtracted from the PSTH to the AB sequence. The obtained activation rates were plotted as for the left subfigures. (**c**) Top: Color-maps of normalized activation rates from 96 units vertically ordered according to their best delays in response to the B sequence. Bottom: Pooled activation rates from all units. (**d**) Suppression rates calculated from responses to dual- and single-object sequences at specific time ranges. The response to the second half of the sequence (450 ms – end of the sequence) is suppressed when echoes from object A are present (sign test < 0.001; left boxplot). The presence of object B echoes has no effect on the response to object A when considering the time range where object A is presented (start – 450 ms; right boxplot; sign test: p > 0.05). The whiskers of the boxplots represent 5–95% percentile. (**e**) Time course of and recovery from suppression calculated with the normalized suppression rates from each unit in a bin-wise manner. Recovery occurred 240 ms after passing object A and it is defined as soon as the suppression rates did not differ significantly from 0 (sign test: p > 0.01 = no suppression).

To visualize the impact of object A on the neuronal tuning, the post-stimulus time histogram (PSTH) to the object B sequence was subtracted from the PSTH to the dual-object sequence containing echo information from object A and B (Fig. 3a). The obtained values were exemplarily plotted as activation rates into a line plot for one unit (Fig. 3b, *top*) but could also be transferred and plotted into a color-map (Fig. 3b, *bottom*). Note that bins showing no difference between the two PSTHs are indicated as white bins in the colormaps. Negative values indicate suppressive events while positive values indicate excitatory effects of object A on the response to the AB sequence. For getting an overview of the impact of object A on the neuronal tuning in each recorded unit, the normalized PSTHs were analysed and the normalized activation rates were plotted into a color-map (Fig. 3c, *left color-map*). Each unit is represented in one line and they are vertically ordered according to their delay tuning from 16 ms to 1 ms. For pooling the data, the median of the normalized activation rates from all units were plotted in a bin-wise manner at the bottom of Fig. 3c. Excitatory effects of object A on the response to the AB sequence were widely spread from the start until the 450 ms mark (black arrowheads in Fig. 3b,c). This is not surprising because in that time window echo information from object A was present in the AB sequence and processed by the units. Suppressive effects were prominent directly after the excitatory responses to object A. The time point of the suppression depended on the delay tuning of the units and occurred later in short- than in long-delay tuned units. For quantifying the suppression strength induced by object A, suppression rates were calculated by taking the ratio between the total amount of spikes in response to the AB sequence and the B sequence in the time window starting at the 450 ms mark. The obtained ratio was subtracted from 1 (Fig. 3d, *left boxplot*). The result of this calculation showed that in 89% (85 out of 96) of the units the response to object B was suppressed by the presence of leading echoes from object A and showed suppression rates above 0. The median suppression rate was significantly higher than 0 (median = 0.38; IQR = 0.22–0.58; sign test: p < 0.001).

To investigate the impact of object B on the response to the AB sequence, the normalized response to the A sequence was subtracted from the normalized response to the AB sequence and the values were plotted as a color-map (Fig. 3c, *right color map*). For an exemplary unit see Fig. 3b. Based on the pooled data, object B had no pronounced suppressive impact on the response to the AB sequence. The absence of suppression is reflected by the suppression rates calculated in the time window from the start to the 450 ms mark (Fig. 3d, *right boxplot*). The suppression rates did not deviate significantly from 0 (median = 0.02; IQR = −0.17–0.33; sign test: p > 0.05), which indicates that echoes from B had no impact on the neuronal response to object A. After the 450 ms mark, excitatory effects induced by the presentation of object B echoes were detected (Fig. 3c, *right color-map*). Note that echoes from object B at that time window were absent in the object A sequence.

Next, we wanted to assess the time course of and recovery from suppression that is induced by object A. The number of spikes from the 450 ms mark until the end of the sequence in response to the object B sequence was subtracted from the spike count evoked by the AB sequence in a bin-wise manner. For each unit, the resulting spike-count differences obtained for each bin were normalized to the maximum absolute difference and averaged to create a pooled suppression rate for each 5 ms bin (Fig. 3e). The results of this calculation showed that right after passing object A (time point 0 in Fig. 3e) the suppression was maximal, as indicated by suppression rates close to 1. The suppression decreased over time reaching values that were not significantly different from 0, starting at ~240 ms after passing object A (sign test: p > 0.01).

### Impact of each object on the neuronal response

**Figure 4:**
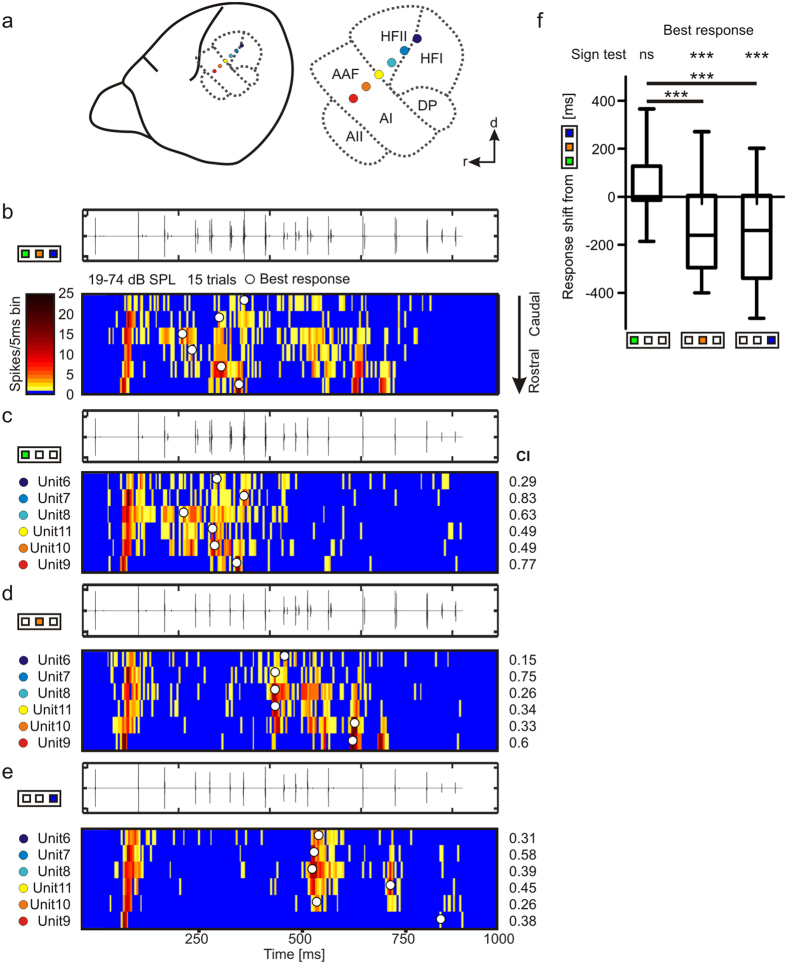
Responses to multiple- and single-object sequences of six units recorded simultaneously within the chronotopically organized cortex regions. (**a**) Schematic lateral view on *C. perspicillata*’s brain and magnified auditory cortical areas (dashed lines). Colored spots denote electrode positions. The linear electrode array was positioned slightly oblique along the rostro-caudal axis and covered part of the high frequency areas end extended into the high frequency regions of the primary auditory cortex. *d* = dorsal, *r* = rostral. (**b**–**e**) For each stimulus the oscillogram (upper) and activity pattern of six units in response to the ABC (**b**), A (**c**), B (**d**), and C (**e**) sequences are shown in a color-map with a binsize of 5 ms. Each row represents the activity pattern from one unit in response to 15 trials. The units were recorded simultaneously from the auditory cortex and their positions follow the chronotopy along the caudo-rostral axis of the cortex (**a**). White dots in color-map mark the time point of best responses. The correlation indices (CIs) are indicated at the right side. In five out of six units, CIs were higher between the response pattern to the A sequence and the multiple-object sequence than between responses to the B or C sequences and the multiple-object sequence. (**f**) Response shifts for the best response mediated by each object are plotted against each other. Respectively, positive or negative shifts indicate that the response occurs later or earlier in the multiple-object sequence than in the corresponding single-object sequence. Best response differed minimally between multiple-object sequence and the A sequence. Best response differed more strongly between multiple-object sequence and the B or C sequences. Thus, the delay tuning in response to the multiple-object sequence is mostly determined by object A and less by object B and C. Kruskal-Wallis and Dunn’s multiple comparison post hoc test (***p < 0.001).

After characterizing the suppression, we wanted to quantify the relative impact of each object on the response pattern to the multiple-object sequence that contained echoes from the three objects. For each unit, the time point of the best response was calculated in response to the ABC sequence and to each of the single-object sequences. At the cortical level, delay-tuned neurons are topographically organized in some bat species, including *C. perspicillata*^8^. In the dorsal auditory cortex of *C. perspicillata*, neurons tuned to short delays are confined to rostral regions while neurons tuned to long delays appear mostly in caudal regions. A representative response pattern from six units recorded simultaneously with an electrode array positioned along the chronotopic gradient of the dorsal auditory cortex of *C. perspicillata* is shown in Fig. 4. Each row of the color-maps represents one unit recorded at a specific cortical location (denoted by dot color; Fig. 4a). Note that the *orange* unit in Fig. 4 is the example unit shown in Fig. 3a,b. According to the neuronal activity in response to the B sequence (Fig. 4d), the caudal units (unit 6, unit 7, unit 8, and unit 11) had best delays in the range of 7–9 ms, whereas the two remaining rostral units, unit 10 and unit 9 had best delays between 4 and 5 ms. When comparing the neuronal response to the multiple-object sequence (Fig. 4b) with the response to the single-object sequences (Fig. 4c–e), it is evident that the time points of the best responses (*white dots* in Fig. 4) in the object A sequence are close in time to the best responses observed in the multiple-object sequence. In contrast, the time points of best responses in the two remaining single-object sequences differ vastly from the time points of best responses in the multiple-object sequence.

The same results can also be seen in the pooled data (Fig. 4f). Differences of best responses (response shifts) are significantly smaller between the multiple-object sequence and object A sequence than between the multiple-object sequence and the B or the C sequences. Based on these observations one could propose that the response to the multiple-object sequence is strongly shaped by echoes from object A. The same results can also be seen for processing the dual-object sequences (Supplementary Fig. 3).

**Figure 5:**
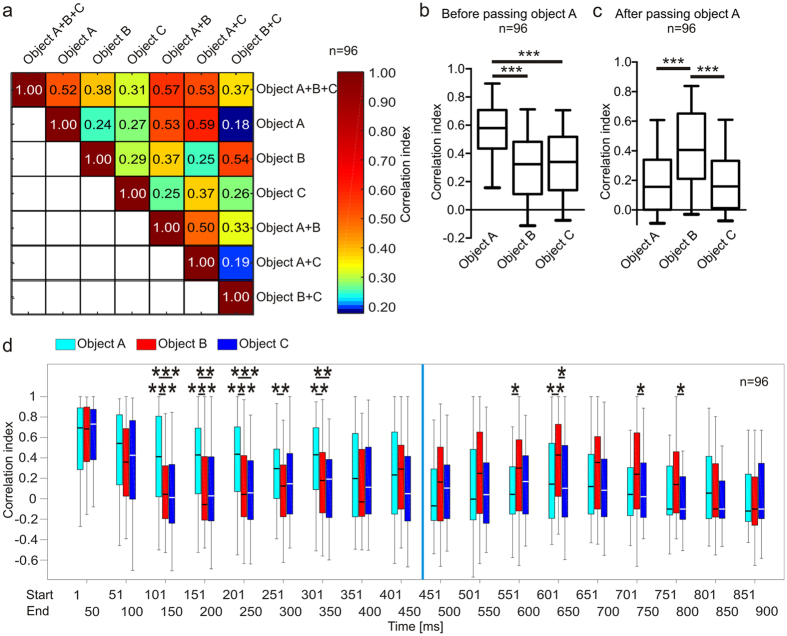
Neurons focus on spatially closest objects. (**a**) Pooled correlation indices from PSTHs in response to multiple-, dual- or single-object sequences. (**b**) Correlation indices between PSTHs of each single-object sequence and to the multiple-object sequence before passing object A. (**c**) Correlation indices between PSTHs to each single-object sequence and to the multiple-object sequence after passing object A. (**d**) Time course of correlation indices calculated from 50 ms time windows of the PSTHs of each single-object sequence correlated to the corresponding time windows in the multiple-object sequence. Each 50 ms time window consisted of ten 5 ms bins of the PSTHs. Note that before passing object A the PSTH in response to the A sequence mostly resembles and thus has highest impact on the response pattern to the multiple-object sequence (indicated by significantly higher correlation indices for *cyan* boxplots). After passing object A the PSTHs in response to the B sequence mostly resembles the PSTH in response to the multiple-object sequence (indicated by significantly higher correlation indices for *red* boxplots; Kruskal-Walis and Dunn’s multiple comparison post hoc test; *p < 0.05; **p < 0.01; ***p < 0.001).

To take the overall activity pattern into account, the PSTH obtained in response to the multiple-object sequence was correlated with the PSTHs to each single-object sequence. The higher the correlation indices (CIs), the more similar are the compared PSTHs. In the example units from Fig. 4, and at the population level (Fig. 5a) the CIs between the single-object sequences and the multiple-object sequence were maximal when comparing the response to the multiple-object sequence with the response to the A sequence (see CI values at the right corner of Fig. 4c–e). Thus, the PSTHs to the A sequence are the ones that resemble more the PSTH obtained in responses to the multiple-object sequence. These results suggest that in a multi-echo environment, responses are mostly determined by the first-arriving echo, in this case the echo from object A, while the responses to later echoes are either absent or partly suppressed. This idea is further confirmed by the fact that before passing object A (start of sequence until 450 ms mark) CIs were highest between PSTHs obtained in response to the A sequence and PSTHs obtained when playing the ABC sequence (Fig. 5b). After passing object A (after 450 ms mark until the end of the sequence), a situation in which the echoes from object B are leading, the response to the B sequence led to relatively high CIs while the CIs obtained from the C sequence were similar to those obtained from the A sequence, even though echoes from object A were absent after 450 ms into the sequence (Fig. 5c). Note that, at the population level, the neuronal response to the multi-object sequence was more similar to the response to the dual-object sequences containing echo information from object A (CIs = 0.57 and 0.53, respectively) than to any response to the single-object sequence (CIs = 0.52; 0.38 and 0.31; Fig. 5a). This result reflects the physical similarity between the acoustic stimuli. The dual-object sequences contain more echoes from the multiple-object sequence than the single-object sequences, and the latter is reflected in the response patterns.

In a more detailed temporal analysis, the CIs were calculated with 50 ms bins where ten 5 ms bins from the PSTHs to the ABC sequence were compared with the corresponding bins from the PSTHs to the single-object sequences. Here, at the beginning of the stimulation, the CIs were relatively high for each object but after 100 ms it becomes evident that object A strongly drives the activity pattern in response to the ABC sequence (Fig. 5d). The high CIs at the beginning of the sequence can be explained based on strong initial responses to acoustic stimuli a phenomena that has already been described in a previous study^14^. From 400 ms on (directly before object A is passed), the CIs from object B increased until being significantly higher than the CIs from the A or C sequence at the time windows 551–600 ms. It could be that at that time point, enough neurons had already recovered from the suppression caused by object A, and thus the response to the object B sequence resembles best the response to the multiple-object sequence. Note that the impact of object C was relatively low throughout the stimulation in the multiple-object sequence, which could point towards a forward suppression that was induced by object B after the 450 ms mark.

## Discussion

In their natural environments, animals have to cope with multiple stimuli arriving simultaneously or sequentially to their sensory organs. Not each stimulus has the same behavioral relevance. Therefore, it would be expected that stimuli are processed differentially in the brain depending on their ethological value. Studies in vertebrates and invertebrates have revealed that animals focus their visual gaze in a complex scenario and that they change their focus from time to time through saccadic movements^2^,^18^,^19^,^20^,^21^,^22^,^23^,^24^. Behavioral experiments have already demonstrated that echolocating animals, like bats and toothed whales, fixate the acoustic gaze through spatially focusing their sonar beam^6^,^10^,^25^,^26^,^27^,^28^,^29^,^30^,^31^,^32^,^33^. The latter has been interpreted as a mechanism that attempts to reduce the amount of echo reflections produced by surrounding objects. A recent study in the species *Phyllostomus discolor*, a close relative to *C. perspicillata*, showed that the spatial focus of the sonar beam seems to be extremely dynamic and the restriction is facultative for the animals^34^. It appears that in bats spatial restriction of the sonar beam is not mandatory for proper orientation. Therefore, it is unlikely that in target-rich environments only one echo is produced per biosonar call emission, even if the bats restrict their sonar beam. Note that the multiple echoes derived from each call emission are not overlapping in time, and therefore they do not create spectral notches that can be used for μs time estimations, as describe for *Eptesicus fuscus*^35^,^36^. We discuss three scenarios on how echo information from multiple objects can be processed in the brain of echolocating bats. (i) Echo information from all objects is processed in multiple streams in the cortex (parallel processing). Theoretically, if the distance between two objects is long enough, then short delay-tuned neurons could process the target distance from the nearest object, whereas echoes from more distant objects could be processed by long-delay tuned neurons. (ii) Echo information from one object is preferentially processed (single object processing). (iii) Not the distance between the animal and the object is processed, but the distance between multiple objects is processed with the help of the delay between consecutive echoes. Note that processing the distances between objects would be less beneficial to the animal, assuming that the bat’s main goal is to determine target distance to avoid collisions. However, it may have advantage for complex scene analysis.

Delay tuning depends on an interaction between excitation and inhibition^11^,^37^. The broadcasted call usually evokes an inhibition followed by a rebound excitation. If rebound excitation triggered by the call coincides with an excitation in response to the echo, then firing occurs in the delay tuned neurons. Therefore, a preceding call opens a temporal integration window for processing echo information. Because of the weakened response to second or third echoes described in the present study, one could suggest that the most sensitive temporal processing window is closed after first echo arrival.

A processing strategy based on parallel processing (Hypothesis 1) provides the animal with the most detailed distance information about its surrounding. However, this coding strategy may not be ideal for initiating fast motor responses because the cortex has to cope simultaneously with multiple processing streams. A coding strategy that focuses on distance information from one specific object would be simpler, and our results show that cortical responses to multiple-object sequences are essentially determined by responses to leading echoes. Responses to lagging echoes are subjected to suppression. Thus, there seems to be a “default” preference in the neuronal tuning to close objects by cortical units that is robust and evident even in anaesthetised animals. In other words, cortical suppression does not only increase the sharpness of delay-tuning as demonstrated in previous studies^14^,^38^,^39^,^40^ but it might also help the animal to focus by default on near obstacles while flying in multi-object environments. Parallel processing and processing distances between objects are unlikely to be realized without selective attention that may be used to allow a correct identification of an auditory event to be a call or an echo. Without such processing taking place, cortical neurons appear to define by default the first auditory event as a call and the second event as an echo. Echoes occurring shortly afterwards are largely subjected to cortical suppression, which also prevents a misdefinition of call and echo.

The presence of cortical suppression avoids parallel processing of multiple auditory streams. A parallel processing strategy has been proposed by electrophysiological studies that used simple single call/multiple echo elements^39^,^41^. Suppressing distance information from lagging echoes occurs at the expense of losing information from distant targets. However, one has to keep in mind that this suppression could be of advantage for the generation of fixed motor patterns important for rapidly moving animals that, like bats, have to orientate in permanently changing and complex environments. In other words, focussing cortical neuronal responses by default on the nearest object could help the animal to avoid crashing into an immediate obstacle during flight. This does not mean that during echolocation, echoes from distant objects are not processed. Behavioral studies showed that after cortical ablation, bats are still capable to avoid collisions during flight^42^. A phenomena that is abolished after bilateral ablation of ventral parts of the inferior colliculus^43^. How subcortical structures like the inferior colliculus process the echolocation streams of the present study should be addressed in future studies. Additionally, for long range orientation bats could rely on vision and memory for planning their flight path^44^,^45^,^46^. This memory could be adjusted through delay information coming from near objects. Finally selective attention could additionally affect neuronal tuning by acting directly on the neurons or by changing the amount of information that reaches the cochlea via the efferent system^47^,^48^ or changes in pinna position and/or in the stiffness of the middle ear^49^. Different behavioral adjustments can change the sensory world that bats are facing or perceiving. During call emission, the animals can variably adjust their sonar beam^49^ and the echo perception can be influenced through motor behaviors of the head or the pinnae^49^,^50^. A recent study on insect-eating bats demonstrated that, while hunting, the bats shift their sonar beam and flight path towards the second prey before capturing the immediate prey which increases the capture rate^51^. In the present study, the effects of attention could not be tested because neuronal recordings were performed in anesthetized bats. However, it is possible that the attention of the animal that was swinging in the pendulum for stimulus recordings could be represented on the call parameters of the echolocation sequence. It is known that other bat species like *E. fuscus* actively adjust their sonar parameters when in multiple object environments to focus on objects that are far away^6^,^10^. Such behaviour has not been demonstrated in fruit eating bats, such as the one studied here, and therefore whether and how it influences neuronal responses remains, at present, unknown.

In conclusion which impact top-down mechanisms like attention or behavioural state of the animal has on the coding strategy remains speculative but it is noteworthy, that even without selective attention the animal could process target-distance information from the nearest obstacle.

## Methods

### Animals

Electrophysiological experiments were conducted in six adult bats (5 females and 1 male) of *Carollia perspicillata*. The bats were bred in a colony of the Institute for Cell Biology and Neuroscience (Frankfurt University). The animal use in the experiments complies with all current German laws on animal experimentation and it is in accordance with the Declaration of Helsinki. All experimental protocols were approved by the Regierungspräsidium Darmstadt (experimental permit #F104/57).

### Stimulus recordings, constructions and presentations

For recording a natural echolocation sequence that was used later on as acoustic stimulus on anesthetized bats, a bat was placed in a pendulum^12^,^13^,^14^. An ultrasound sensitive microphone (Avisoft Bioacoustics, Germany) was medially positioned above the animal’s head and adjusted as close as possible to the ears (~4 cm). The microphone had a sensitivity of 50 mV/Pa and an input-referred self-noise level of 18 dB SPL. It was connected with a sound acquisition system (UltraSoundGate 116 Hm mobile recording interface, +Recorder Software, Avisoft Bioacoustics, Germany) for sound digitalization at 375 kHz (16 bit precision). The bat was swung (total distance = 4 m) and it faced three objects that were separated along the depth axis during the swing. Object A was a dummy rock (depth: 65 cm; width: 95 cm; height: 35 cm) made out of papier-mâché and it was overflown by the animal before the swing stopped in front of object B, a wooden plate (depth: 0.8 cm; width: 21 cm; height: 21 cm), which was positioned 130 cm after object A. 20 cm behind object B was object C an acrylic glass wall (depth: 0.3 cm; width: 50 cm; height: 150 cm). During the swing the animal broadcasted sequences of calls which were recorded. The acrylic glass wall covered a larger area than the wooden plate, thus echoes coming from both objects were recorded.

Background noise of the stimuli was filtered via “Noise Reduction” (FFT length 256; precision 16) with the software Avisoft SAS Lab Pro (Avisoft Bioacoustics, Germany) as described in ref. 14. To transform the multiple- into single- and dual-object sequences we manually filtered with the software BatSound (PetterssonElektronik AB, Sweden) the echoes belonging to specific objects.

During electrophysiological recordings, acoustic stimuli were played at a sampling rate of 384 kHz (32 bit precision) with an Exasound E18 sound card (ExaSound Audio Design, Canada). The audio signals were transferred to an audio amplifier (Rotel power amplifier, RB-850). The bat was stimulated with a calibrated speaker (ScanSpeakRevelator R2904/7000, Avisoft Bioacoustics, Germany) located at 15 cm from the bat’s ear. The calibration curve was calculated with a ¼-inch Microphone (Brüel&Kjaer, model 4135, Denmark) which was connected to a custom-made microphone amplifier.

The echolocation sequences were played randomly with 15 averages and at intervals of 400 ms. Three different intensity levels were used. The maximal intensity level ranged between 29–84 dB SPL (Supplementary Table 1) and the sequences were attenuated 10 dB and 20 dB for the remaining two sound pressure levels.

### Data acquisition and analysis

Electrophysiological recordings took place in a sound-proofed and electrically-shielded chamber. Neuronal responses were recorded from both brain hemispheres. For anaesthesia, bats were subcutaneously injected with a mixture of ketamine (10 mg × kg^−1^ Ketavet, Pharmacia GmbH, Germany) and xylazine (38 mg × kg^−1^ Rompun, Bayer Vital GmbH, Germany). Surgery and chronical recordings were done as described in ref. 14.

Recordings were performed with custom-built glass electrode arrays of up to 6 channels organized in a row. Glass electrodes (resistance 1–10 MΩ when filled with 3 Mol KCl) were drawn from borosilicate capillaries (GB120F-10, Science Products, Germany) with a Flaming/Brown horizontal puller (P97, Sutter, USA) and they were glued together in a fanshape pattern, thus ensuring an electrode tip space of 250 μm. Penetration occurred with the dura mater remaining intact. The glass electrode arrays were positioned in the high frequency area of the auditory cortex^52^ along the chronotopic gradient^15^. The high frequency area was located caudal to a blood vessel within the depression of the pseudocentral sulcus^15^. Along the dorso-ventral axis the electrodes were positioned 1–2 mm lateral from the scalp midline. The orientation of the electrode array was adjusted in parallel to the scalp midline. Based on frequency-level receptive fields, all analysed multi-units were sensitive to high frequencies. Frequency tuning was assessed with pure tone stimuli of 2 or 10 ms duration (0.5 ms rise-fall time). Tested frequencies ranged from 5–95 kHz and the sound pressure levels were between 30–90 dB SPL. Sound levels were adjusted based on the speaker’s calibration curve. Each frequency-level combination was randomly presented five times with a 400 ms interstimulus time interval. All multi-units sensitive to high frequencies were delay-tuned. Delay-tuning was assessed with a stimulus protocol that allowed a rough calculation of a delay-tuning curve as described in ref. 14.

Neuronal data acquisition used a wireless multichannel recording system (Multi Channel Systems MCS GmbH, Germany), at a sampling rate of 20 kHz (per channel) and 16 bit precision. Neuronal responses were analysed in 96 multi-units. Spike events were detected with a multi-unit specific threshold that was based on the spike amplitude. For each multi-unit, spike threshold was kept constant during the stimulation protocol thus ensuring that the same multi-unit activity was recorded for each stimulus.

All analyses are based on post-stimulus time histograms (PSTHs) used a binsize of 5 ms. Sign tests were calculated in Matlab 2014 and remaining statistics in GraphPad Prism 5 (GraphPad Software, USA). *p < 0.05; **p < 0.01; ***p < 0.001. Only the best intensity for each unit represented by the highest number of evoked spikes in response to the multiple-object sequence was analysed. The time window for best response calculations were from 150 ms to the end of the sequence. The first 150 ms were not considered because strong initial responses often occurred which were not related to delay tuning. To test the similarity between the PSTHs calculated based on the neuronal responses to the multi-, dual- and single-object sequences a correlation analysis was done in Matlab 2014. The higher the similarity, the higher the obtained correlation indices. Correlation calculations were done within different time windows of the PSTHs. For rough overviews a correlation was done through taking the entire PSTH length into account. For detailed correlation analysis the first 450 ms of the PSTHs (before passing object A), after the 450 ms mark (after passing object A) or in 50 ms bins were analysed. The analysis in 50 ms time windows took ten 5 ms bins of the PSTHs into account for the calculation of correlation indices.

## Additional Information

**How to cite this article**: Beetz, M. J. *et al.* Cortical neurons of bats respond best to echoes from nearest targets when listening to natural biosonar multi-echo streams. *Sci. Rep.* **6**, 35991; doi: 10.1038/srep35991 (2016).

**Publisher's note**: Springer Nature remains neutral with regard to jurisdictional claims in published maps and institutional affiliations.

## Supplementary Material

Supplementary Information
