# Supplementary material for: Cortical neurons of bats respond best to echoes from nearest targets when listening to natural biosonar multi-echo streams
Source: Sci Rep. 2016 Oct 27;6:35991. doi: 10.1038/srep35991 (PMC5081524; doi:10.1038/srep35991)
Supplement: Supplementary Information [file srep35991-s1.pdf]

***Title*: Cortical neurons of bats respond best to echoes from nearest targets when listening to natural biosonar multi-echo streams**

Authors: M. Jerome Beetz (*) [1], Julio C. Hechavarría [1], Manfred Kössl[1].

Affiliations: [1.] Institut für Zellbiologie und Neurowissenschaft, Goethe-Universität, Frankfurt/M., Germany.

* Corresponding author

Mailing address:

M. Jerome Beetz

Email: Jeromebeetz@arcor.de

Institut für Zellbiologie und Neurowissenschaft, Max-von-Laue-Straße 13, 60438, Frankfurt/Main, Germany, Tel.: +49 69798 42066

***Short Title*: Cortical responses to natural multi-echo streams**

Table S1 Sequence paramters (refers to Fig. 1)

Calculations were done with Avisoft SASLab Pro (Avisoft Bioacoustics, Germany).

BW = bandwidth; dur = duration; duty cycle = call duration/call interval; f = frequency; int = intensity; rms = root mean square

Polyobject sequence														
# call	dur	Pulse interval	1st delay	2nd delay	3rd delay	int pulse	int 1st echo	int 2nd echo	int 3rd echo	duty cycle	peak f	min f	max f	BW
	[ms]	[ms]	[ms]	[ms]	[ms]	rms [dB SPL]	rms [dB SPL]	rms [dB SPL]	rms [dB SPL]	[%]	[kHz]	[kHz]	[kHz]	[kHz]
1	1.27		11	18.6		76.99	55	54		1.34	82.6	77.4	90.2	12.7
2	1.41	94.45	8.5	16		80.89	62	61		2.46	84.9	79.9	91	11.1
3	1.37	57.37	7.3	15		80.65	67	62		2.03	86.8	81.8	91.9	10.1
4	1.39	67.58	5	10.7		75.66	75	42.5		4.41	71.9	82.1	92.9	10.7
5	1.41	31.52	4	9.3		78.87	79	50.63		3.19	72.7	76.2	93.6	17.3
6	1.62	44.22	3	9.2		78.02	80	45.47		5.47	76.2	83.6	91.4	7.7
7	1.33	29.6	2.3	8.9		78.1	79.4	41.01		2.78	66	80.4	92.8	12.3
8	1.31	47.83	1.7	8.7	14.2	81.5	71.6	65.3	28.95	3.23	60.3	80.3	94.1	13.7
9	1.22	40.52		7.7	13	75.27		67.6	52.1	4.72	84.4	78.6	95	16.3
10	1.1	25.83		7	12.2	76		68.9	52.5	4.19	84.8	79.9	92.4	12.5
11	1.37	26.25		6.5	11.6	77.42		73.5	57.6	3.00	63.6	81.1	95.8	14.6
12	1.77	45.64		5.5	11	80.58		70.5	58.1	2.34	65.1	80	92.3	12.3
13	1.43	75.56		4	9	81.08		78.1	44.87	2.02	86.9	84.2	94	9.8
14	1.33	70.79		3.2	8.7	80.31		83.3	56.98	1.92	83.8	73	93	20
15	1.06	69.43		1.5		82.33		83.7		3.22	83.8	74.7	94.3	19.5
16	0.77	32.91		1.3		72.8		74.5		2.59	79.2	75.8	91.6	15.7
17	0.81	29.75		1		75.68		76.2			76.5	70	92.8	22.7

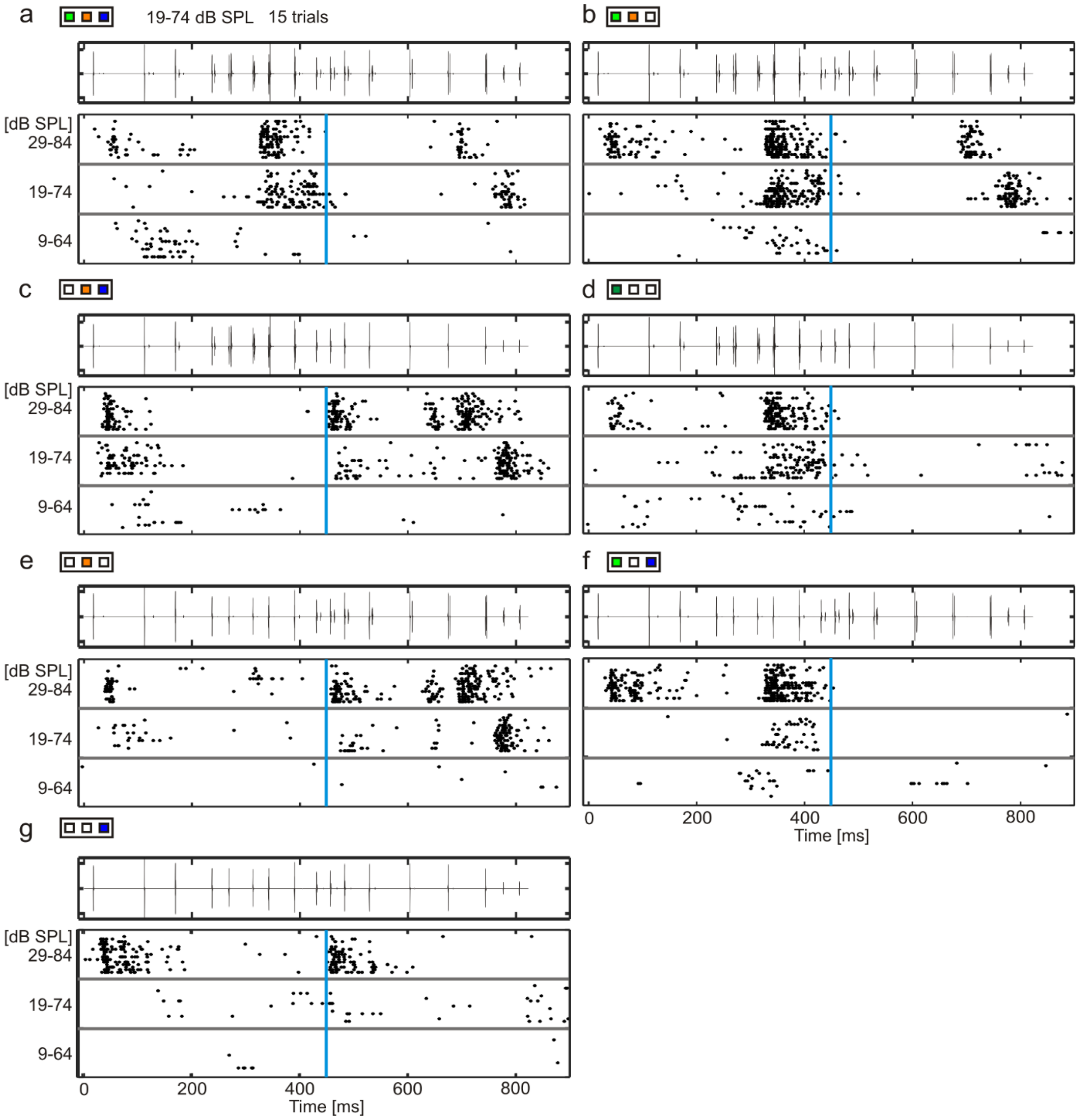

Figure S1. Responses of one unit to different single- and dual- and multiple-object sequences played at three different attenuation levels (refers to figure 2).

For each stimulus the oscillogramm (upper) and raster plot in response to all three attenuation levels are shown. Vertical blue line indicate the time point where echoes from object A disappear due to the bat leaving that object behind in the flight trajectory.

(a-g) Response to object ABC (a), .AB (b), BC (c), A (d), B (e), AC (f) and C (g) sequence. Note that echolocation sequences (b-g) were created through filtering manually the corresponding echoes from sequence (a).

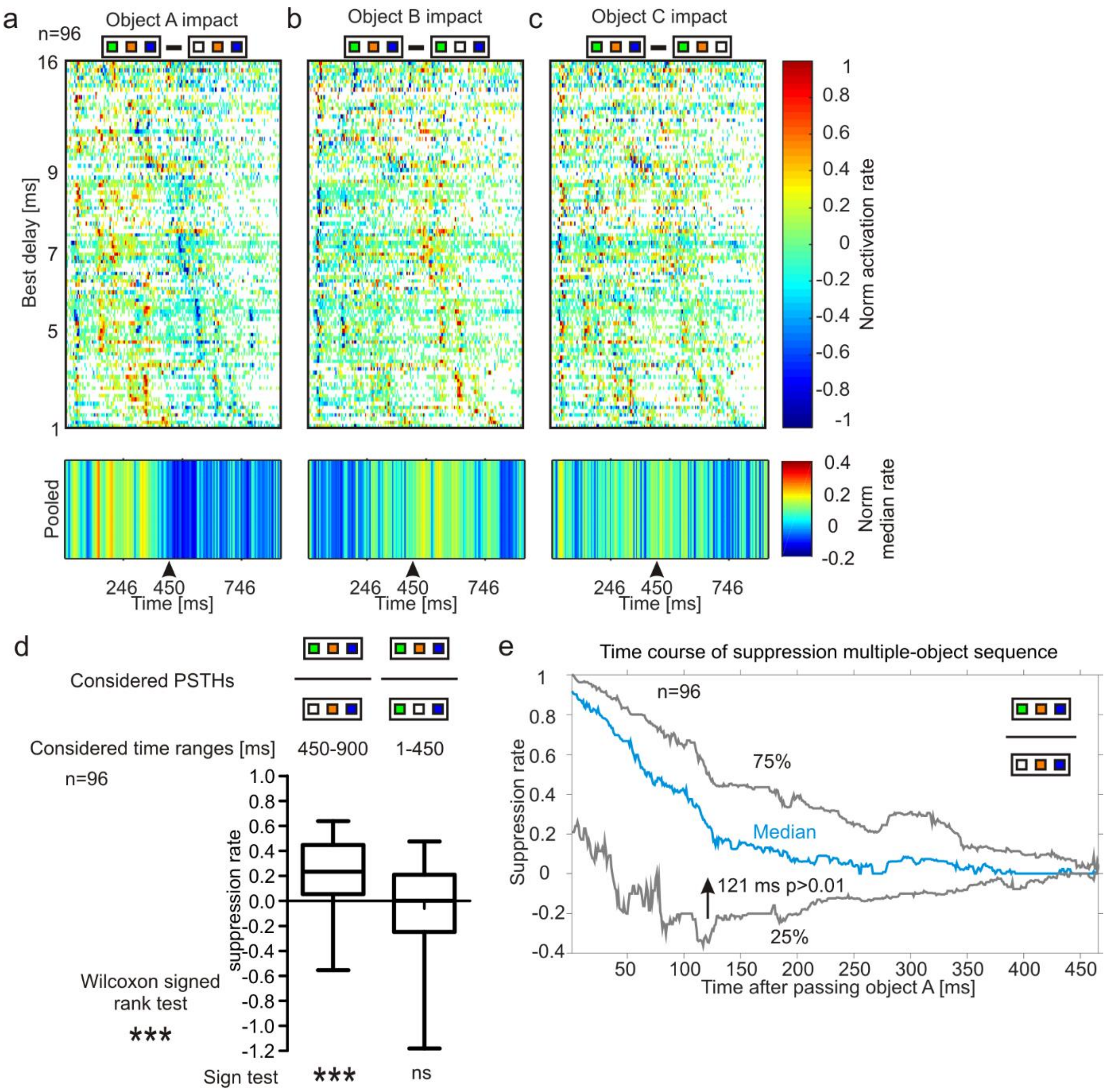

Figure S2. Quantification and time course of suppression when stimulated with the ABC sequence (refers to figure 3).
(a-c) Top: Color maps of normalized activation rates from 96 units vertically ordered according to their best delays in response to the B sequence. Activation rates were calculated through subtracting the PSTHs in response to the BC (a), AC (b) and to the AB (c) sequences from the PSTHs in response to the ABC sequence. Bins, with no difference between the response to the ABC sequence and to the dual-object sequence are white. The activation rates show the relative impact of each object to the overall response along the temporal axis. Negative values indicate suppressive and positive values excitatory impacts of the corresponding object on the response to the ABC sequence. Bottom: Normalized median activation rates from all units. Black arrowheads signal time point of passing object A. Note that after passing object A the response to object B is suppressed in response to the ABC sequence in comparison to the response to the BC sequence (a). Object B and object C had respectively, slight or no suppressive impact on the response to the ABC sequence (b and c). (d) Suppression rates calculated from responses to the ABC sequence and to the dual-object sequences under consideration of specific time windows. Response to object A in the first time window (1-450 ms) is followed by suppression in the second time window (450-900 ms; signtest < 0.001; left boxplot). The presence of object B echoes had no effect on the response to object A in the first time window (right boxplot; sign test: ns). (e) Time course of suppression and recovery range calculated with the normalized suppression rates from each unit and bin for the three object situation. Recovery occurred as soon as the values did not differed significantly from 0 (sign test: p > 0.01 = no suppression) and is indicated by a black arrow.

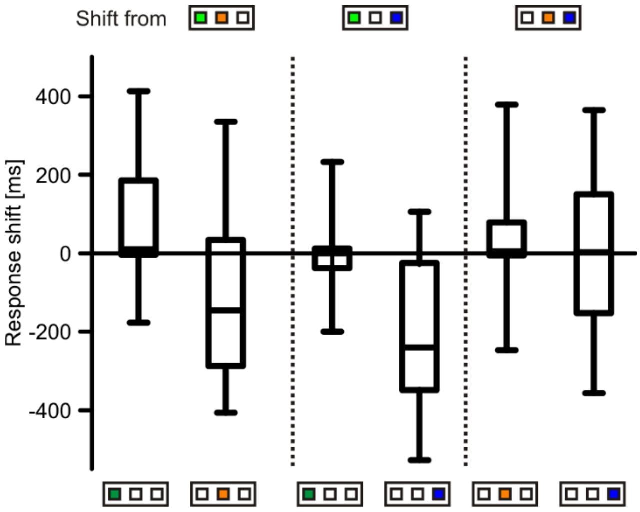

Figure S3. Shifts in time points of best responses induced by cortical suppression in the dual-object sequence (refers to Fig. 4).

Best response shifts between the dual- and single-object sequences. Note that in comparison to the data from figure 4, the response shifts are less between the leading echo and the dual-object sequence, indicated by response shifts that are closer to 0 and smaller quartile ranges than between the lagging echo and the dual-object sequence.
